# A Test of Feasibility and Acceptability of Online Mindfulness-Based Stress Reduction for Lesbian, Gay, and Bisexual Women and Men at Risk for High Stress: Pilot Study

**DOI:** 10.2196/15048

**Published:** 2019-08-16

**Authors:** Jennifer M Jabson Tree, Joanne Gayle Patterson

**Affiliations:** 1 Department of Public Health University of Tennessee Knoxville, TN United States

**Keywords:** sexual minority, lesbian, gay, bisexual, psychological stress

## Abstract

**Background:**

In conservative and rural areas, where antidiscrimination laws do not exist, lesbian, gay, and bisexual (LGB) people are at risk for excess stress arising from discrimination. Stress-reducing interventions delivered via innovative channels to overcome access barriers are needed.

**Objective:**

This study aimed to investigate the feasibility and acceptability of online mindfulness-based stress reduction (OMBSR) with LGB people in Appalachian Tennessee at high risk for stress.

**Methods:**

In 2 pilot studies involving pre-post test designs, participants completed 8 weeks of OMBSR, weekly activity logs, semistructured interviews, and surveys of perceived and minority stress.

**Results:**

Overall, 24 LGB people enrolled in the study and 17 completed OMBSR. In addition, 94% completed some form of mindfulness activities daily, including meditation. Participants enjoyed the program and found it easy to use. Perceived stress (Cohen, perceived stress scale-10) decreased by 23% in women (mean 22.73 vs mean 17.45; *t*10=3.12; *P*=.01) and by 40% in men (mean 19.83 vs mean 12.00; *t*5=3.90; *P*=.01) between baseline and postprogram. Women demonstrated a 12% reduction in overall minority stress (Balsam, Daily Experiences with Heterosexism Questionnaire) from baseline to 12-week follow-up (mean 1.87 vs mean 1.57; *t*10=4.12; *P*=.002). Subscale analyses indicated that women’s stress due to vigilance and vicarious trauma decreased by 21% and 20%, respectively.

**Conclusions:**

OMBSR may be a useful tool to help LGB people reduce general and minority-specific stress in socially conservative regions lacking antidiscrimination policies.

## Introduction

### Background

Lesbian, gay, and bisexual (LGB) people in Appalachian Tennessee hold intersecting identities that make them different from non-Appalachian heterosexual people and LGB people in urban areas. Specifically, intersecting Appalachian and minority sexual orientation identities magnify discrimination, stigma, and stress caused by living outside the heterosexual norm.

Appalachia is a diverse rural geographic region made up of 420 counties located in 13 states, including 50 counties in East Tennessee. Appalachia is a medically underserved region, and residents earn very low income, with many residing in economically distressed counties where the median income is 80% of the average US income. Over 16% of Appalachian residents live below the poverty level and, in many counties, up to 20% of Appalachian households are living in poverty [1]. A larger proportion of residents in Appalachia (42%) live in rural areas compared with the rest of the United States (20%) [2].

Like many rural regions, Appalachia is socially a conservative region that upholds traditional values that preserve social traditions and morality that condemns LGB people. People with sexual orientations other than heterosexuality are regarded as perverted abnormalities and are systematically stigmatized, ostracized, and socially isolated [3]. It is also a region that lacks state and local antidiscrimination policies and laws [4], and this reinforces the interpersonal and structural stigmatization of sexual minorities [5]. Yet, Appalachian and sexual minority identities are both extremely important for this group [3,6] and rejecting either would be damaging to their self-concept and well-being.

Relatively little empirical evidence exists about the health of LGB people who live in Appalachian Tennessee. However, it is possible that the risks experienced by Appalachian and LGB people may magnify stress, health risks, and poor health for individuals who are both LGB and reside in Appalachia. For example, in our research, lesbian women in Appalachia experienced 40% higher perceived stress than the published norms and high-risk health behaviors, including tobacco use, physical inactivity, and obesity [7].

Behavioral interventions are needed for reducing stress and improving health in high-risk subgroups, including Appalachian LGB people. Mindfulness-based stress reduction (MBSR) is one promising behavioral intervention to reduce stress. Mindfulness is cognitive training [8-11] in self-regulation of attention and orientation to experience [11]. MBSR programs involve 8 weeks of weekly face-to-face, 2.5-hour group sessions, with a trained facilitator in a clinical setting, in addition to daily at-home formal and informal mindfulness-based activities. MBSR interventions produce clinically meaningful reductions in stress in clinical and nonclinical samples and show twice the stress reduction as other behavioral and cognitive interventions [12].

Owing to the risk for being outed and exposed to discrimination and stigma, Appalachian LGB people’s intersecting identities reduce the likelihood of attending traditional, clinic-based, face-to-face interventions [13]. In addition, LGB people in Appalachia experience numerous logistical barriers including cost of attendance, travel, and time away from work. Therefore, innovative delivery channels and adaptation may be needed to reach and deliver MBSR to this high-need group.

Online mindfulness-based stress reduction (OMBSR) may be a solution to the logistical barriers that may limit Appalachian LGB people’s participation in MBSR. OMBSR interventions can be, and have effects, similar to those delivered by trained facilitators. Participants completing OMBSR have shown a clinically meaningful, greater than or equal to 10%, stress reduction from baseline [14,15]. Reducing stress by 10% or more among LGB people in Appalachia could be enormously beneficial for reducing the risk for stress-related health issues [16].

To date, there is no published evidence concerning the feasibility or acceptability of OMBSR with Appalachian LGB people. As with other vulnerable subgroups, it is possible that LGB people in Appalachia have unique needs and experiences that could impact program enrollment, retention, and completion. It is not known if LGB people in Appalachia will enroll in OMBSR, find it useful, enjoy or complete activities, or require substantive or other adaptations for maximum uptake. If LGB people in Appalachia have unique needs and experiences that impact program enrollment, retention, and completion, determining feasibility and acceptability will provide necessary information to guide how we move forward with randomized efficacy trials and program tailoring.

Including LGB women and men in a single behavioral intervention could be beneficial for stretching limited time and financial resources for interventions and broad intervention dissemination. LGB men and women experience similar sexual orientation–related minority stressors in the form of interpersonal and structural stigma and discrimination; however, these groups may differ in terms of health risks and health-related experiences by gender [17-20]. In addition, there may be gender-based differences in the effectiveness of MBSR in the general population wherein women are more likely to engage in MBSR activities than men [21]. Therefore, investigating gender differences in OMBSR use is important for developing MBSR interventions that maximize program efficacy while minimizing expense.

### Objective

This project investigated the feasibility and acceptability of an 8-week OMBSR program delivered to LGB women and men in Appalachia. Our main objectives were to determine the (1) acceptability and amount and aspects of OMBSR that could be delivered to and completed by LGB people residing in Appalachian Tennessee, (2) differences by gender, and (3) preliminary associations between the OMBSR program and perceived and minority stress. To fulfill these objectives, we conducted 2 pilot studies, each using a pre-post test design.

## Methods

### Participant Recruitment

The participants were recruited into an 8-week OMBSR program through a mix of convenience and snowball sampling, an effective strategy for difficult-to-locate populations [5,22]. The inclusion criteria were living in Appalachian east Tennessee; identifying as lesbian, gay, or bisexual; being able to read English; aged 18 years or older; and having internet access. The eligible participants were invited to participate and then each provided informed consent. The informed consent process involved providing participants with detailed project protocol description, including description of the 8-week OMBSR program and data collection activities. LGB people were ineligible if they were diagnosed with thyroid or pituitary gland disorders. The enrolled participants received compensation for survey completion and interviews. All participants provided informed consent before participation. This project was approved by the University of Tennessee Institutional Review Board (UTK IRB-16-02769-FB).

### Online Mindfulness-Based Stress Reduction Procedure

The OMBSR intervention was a free, 8-week OMBSR program [23]. The program content paralleled Kabat-Zinn’s in-person MBSR [11]. The participants logged on to the OMBSR website weekly to receive intervention content and activities. The content included videos and readings about how mindfulness impacts the body and brain and how to apply mindfulness to difficult emotional experiences (Table 1). Formal activities included 10- to 30-min guided meditations. Informal activities involved applying mindfulness principles to daily living (ie, bringing awareness to the moment, nonjudgment, and breathing exercises).

**Table 1:** Weekly content in the 8-week online mindfulness-based stress reduction intervention.

Week	Intervention content		
	Main topic	Formal practice	Informal practice
1	Simple awareness	Body scan	Simple awareness and/or mindful eating
2	Attention and the brain	Introduction to sitting meditation	Pleasant events calendar
3	Introduction to yoga	Mindful Yoga (Yoga 1), body scan, sitting	Unpleasant events calendar
4	Stress: responding versus reacting and 1-min breathing space	Mindful Yoga (Yoga 2) and sitting	STOP: the 1-min breathing space
5	Dealing with difficult emotions and sensations	Various *(soften-soothe-allow meditation on 1st day)*	The soften, soothe, allow process
6	Mindfulness and communication	Body scan, sitting, Yoga *(+ mountain or lake meditation)*	Communication calendar
7	Mindfulness and compassion	Body scan, sitting, Yoga *(+ loving kindness)*	Any (simple awareness, mindful eating, STOP, soften)
8	Conclusion: developing a practice of your own	None	None

### Measures

#### Feasibility and Acceptability

Acceptability was measured with semistructured, qualitative interviews conducted at week 8 after completing the OMBSR program. The questions assessed the participants’ preferred OMBSR activities, skipped and disliked activities, program challenges and successes, requested improvements and changes, and qualitative changes in health and stress.

#### Amount and Aspects of Online Mindfulness-Based Stress Reduction Completed

Self-reported online weekly activity logs measured the amount and aspects of OMBSR completed weekly by participants. Activity logs were specific to weekly intervention content (available upon request). The first 4 questions of the activity log were set to a 4-point Likert scale (3=every day, 0=never) and included questions about activities completed and frequency of practice. Participants rated the usefulness of OMBSR videos, readings, and formal (meditation) and informal (mindful awareness to a routine activity) mindfulness activities on a 5-point Likert scale (5=very useful, 1=not at all useful). The participants scored a zero for activities they did not complete.

#### Perceived Stress

Perceived stress was measured with Cohen’s 10-item perceived stress scale (PSS) [24]. Items were set to a 5-point Likert scale (0=never, 4=very often). Items were summed to generate a PSS score; low scores indicated less perceived stress.

#### Minority Stress

Self-reported experiences with minority stress were measured with the Daily Experiences with Heterosexism Questionnaire (DEHQ) [25]. Items were on a 6-point Likert scale (0=did not happen, 5=it happened and bothered me extremely). Items were averaged across all items and for each subscale; lower scores indicated lower minority stress.

#### Demographic Characteristics

Age, race/ethnicity, education, income, and relationship status were collected using standard questions from the Behavioral Risk Factor Surveillance System [26]. Participants self-reported their sexual orientation with one question asked during eligibility screening.

### Analyses

Descriptive and summary statistics described and compared participants’ demographic characteristics, program completion, and aspects of program completed, stratified by gender.

Qualitative content analyses were conducted on professionally transcribed semistructured interviews [27]. This process involved reading and re-reading the transcripts to achieve immersion. Then, transcripts were re-read for content analysis. Overall, 7 deductive codes were identified before conducting content analyses: device preferences, activity preferences, positive and negative feelings about the program, struggles with the program, positive consequences of the program, and recommendations for program improvements.

Summary and descriptive statistics were calculated on perceived and minority stress measures. Per-protocol and intention-to-treat (ITT) analyses were conducted on perceived and minority stress variables. For ITT analyses, baseline stress values were carried forward for participants lost to follow-up. Paired samples *t* tests tested changes in stress from baseline to postprogram and baseline to follow-up. Repeated-measures analysis of variance tested mean values for each measure of stress against one another at the 3 time points.

## Results

### Participant Demographic Characteristics

A total of 16 lesbian women and 8 gay and bisexual men enrolled in the study; 11 women and 6 men completed the full program and assessments and 5 women and 2 men were lost to follow-up (Multimedia Appendix 1). Multimedia Appendix 1 summarizes the participants’ demographic characteristics; there were no significant differences in demographic characteristics between those who completed and those who did not complete the OMBSR program. Women and men were similar across all demographic characteristics with only one exception—current relationship status. Of program completers, women were more likely to be in a committed relationship than men.

### Feasibility and Acceptability

Most participants reported that the program was easy to use and well organized:

Well yeah, I think it’s convenient where ever I’m at I don’t have to lug around my laptop. I do only have an iPhone 5, so it’s not as big. I do like having it right there. Websites and the links are really easy to navigate, so it’s not hard.

OM101, female participant

I was not in town and the website worked just fine. I was out of the country and it worked just fine, and I got to log on.

OM205, male participant

OMBSR provided enough variety for participants to acquire the instruction needed to feel successful in their mindfulness practice, although their preferences for activities varied. Some participants reported preference for readings or videos, others preferred guided meditations, and some preferred yoga:

I think I’m liking the videos the best, because of the diversity of those, different people, different approaches.

OM110, female

First, I’ll be honest, I did not like the body scan at all, but toward the end of it I found myself doing that more. I guess doing that in the evening sort of prompted that to be one of my favorites because it helps me relax too and settle in for the evening.

OM207, male

Others described the specific activities that they did not like:

For some reason, it’s weird, but I don’t particularly like doing the body scan thing.

OM103, female

Not that I didn’t like the readings and videos, but there were a lot of them to sort through, so I didn’t feel like I could get to all of them throughout the program... There were days I set precedent, or priority to doing the mindfulness practice over watching the video.

OM207, male

Time was the most common barrier to participation; nevertheless, participants reported feeling calmer and less *stressed out*, having greater awareness of the moment and their emotions, and processing experiences differently because of mindfulness activities:

Well, so far, I feel my attention has gotten better. I’m slowing down and I’m on the verge of a panic attack ‘cause I have so much going on that I’m able to stop and slow myself down and focus on something current and right now and quit worrying about five minutes from now. It allows me to be more present.

OM105, female

The program made me feel that I’m not the only one struggling with finding a sense of inner peace, and that many people struggle with the same difficulties I struggle with. And, to just take things one step at a time and center myself around breathing, and that meditation isn’t about trying to eliminate your issues; it’s a way to look deeper into them and cultivate a sense of love for yourself.

OM209, male

Overall, participants were happy with OMBSR as it was presented; only 1 participant expressed that they expected the program to be specifically tailored to LGB people:

If there’s any LGBTQ+ individuals who teach mindfulness courses and have videos and courses on that if those were incorporated in to it, or if there were readings specifically for members of this [LGB] community. Like stress reduction, I feel like that would help a lot because it would be tailored to specific [LGB] experiences and stressors that are in my life right now.

OM206, male

However, women and men indicated that they would like the program to include a social component, and this request varied by gender. Women requested a digital social component (eg, private Facebook group) that would support OMBSR participation in 2 ways: (1) as a collaborative resource for asking questions to fellow participants and researchers about specific readings or activities and (2) as a tool to increase social connectedness between LGB women in the region. Men requested that an in-person social component be integrated into OMBSR to support accountability for daily mindfulness activities and program continuation:

First, because it is difficult to have a community of lesbians, period. It would be nice to be able to talk about the meditation and things that spur from that.

M106, female

Something that I would love—so, I guess, to me, it would be an improvement if there was a way to connect with somebody, or a group of people, that were going through the same process. I would love to just even have somebody to just discuss it with...

OM115, female

I would’ve enjoyed an in-person interaction. It would have been difficult depending on location so maybe the next best thing would be a closed FB group. But there’s a certain sense of accountability that comes from social interaction so I think I would’ve benefitted from that. If people could have said… I really liked this video, you should watch X, or Y video or do this reading.

OM207, male

### Amount and Aspects of Online Mindfulness-Based Stress Reduction Completed

Among participants completing the 8-week program, all reported completing some MBSR practice across the program duration. Women reported completing meditation and/or yoga and informal mindfulness between once or twice and most days (mean 1.55, SD 0.52 and mean 1.73, SD 0.79, respectively). Men reported completing meditation and/or yoga and informal mindfulness practices on most days (mean 2.00, SD 0.00 and mean 2.17, SD 0.41, respectively; Table 2).

**Table 2:** Average type, frequency, and usefulness of participation in online mindfulness-based stress reduction in lesbian, gay, and bisexual women and men, per protocol.

Average characteristics	Women (n=11); mean (SD)	Men (n=6); mean (SD)	*t* (df)	*P* value
Frequency logged on to program website^a^	1.73 (0.47)	2.17 (0.41)	1.93 (15)	.07
Frequency of practicing meditation and/or yoga^a^	1.55 (0.52)	2.00 (0.00)	2.89 (15)	.02
Frequency of informal mindfulness practices^a^	1.73 (0.79)	2.17 (0.41)	1.23 (15)	.23
Usefulness of meditation and/or yoga practice^b^	3.00 (1.26)	4.17 (0.75)	2.05 (15)	.06
Usefulness of informal mindfulness practices^b^	3.09 (2.91)	4.00 (0.63)	2.43 (15)	.03
Usefulness of readings^b^	3.82 (0.75)	4.00 (0.63)	0.50 (15)	.62
Usefulness of videos^b^	3.70 (1.16)	4.17 (0.75)	0.88 (14)	.40

^a^Item measured on 4-point Likert type scale where 0=never, 1=once or twice, 2=most days, and 3=everyday.

^b^Item measured on 5-point Likert-type scale where 1=Not at all useful, 2=somewhat useful, 3=neither useful or not useful, 4=somewhat useful, and 5=very useful.

Significant gender differences were observed. Men reported practicing meditation and/or yoga more than women (mean 2.00, SD 0.00 vs mean 1.55, SD 0.52; *t*_15_=2.89; *P*=.02). Men viewed informal mindfulness activities as more useful than women did (mean 4.00, SD 0.63 vs mean 3.09, SD 2.91; *t*_15_=1.93; *P*=.03).

### Perceived and Minority Stress

#### Women

Per protocol analysis showed that women’s perceived stress average was 22.73 (SD 4.52) at baseline and decreased by 23% (mean 17.45, SD 5.80; *t*_10_=3.21; *P*=.01) at postprogram (Table 3). At the 12-week follow-up data collection, on average, women’s perceived stress was 20% less than baseline (mean 18.09, SD 6.14; *t*_10_=2.49, *P*=.03). As expected, ITT analyses showed similar but less dramatic decreases in perceived stress.

Per protocol, among women, the DEHQ score declined by 12% from baseline to postprogram (mean 1.87, SD 0.37 vs mean 1.65, SD 0.37); this change did not achieve significance (*t*_10_=1.90, *P*=.09; Table 3). However, decline in DEHQ score did achieve significance at 12-week follow-up; among women, daily heterosexist experiences declined 16% from baseline to 12-week follow-up (*t*_10_=4.12 *; P*=.002). As expected, ITT analyses of participants’ report of DEHQ showed similar but less dramatic differences.

Reductions were observed in 2 DEHQ subscales: vigilance and vicarious trauma. Vigilance decreased from baseline to postprogram (mean 2.24, SD 1.08 vs mean 2.00, SD 0.86); this change was not significant (*t*_10_=0.92, *P=*.38*).* However, vigilance significantly reduced by 21% from baseline (mean 2.24, SD 1.08) to the 12-week follow-up (mean 1.77, SD 0.91; *t*_10_=4.20; *P*=.002). Reductions were also observed in vicarious trauma. Significant reductions occurred from baseline to postprogram (mean 4.21, SD 0.91 vs mean 3.38, SD 1.09; *t*_10_=2.69; *P*=.02) and baseline to 12-week follow up (mean 4.21, SD 0.91 vs mean 3.12, SD 1.29; *t*_10_=3.67; *P*=.004). ITT analyses showed similar but less dramatic results for both subscales (Table 4).

#### Men

Per protocol, men’s postprogram perceived stress was 19.83 (SD 4.53) at baseline and decreased by almost 40% at postprogram (mean 12.00, SD 3.58, *t*_5_=3.90; *P*=.01). In ITT analyses, perceived stress decreased by 30% from baseline (mean 19.88, SD 1.37) to postprogram (mean 14.00, SD 1.70; *t*_7_=3.01; *P=*.02 *)* (Table 5).

Neither per-protocol nor ITT analyses indicated any differences in men’s postprogram or follow-up daily heterosexist experiences from baseline (Table 6).

**Table 3:** Within-subject differences in perceived and minority stress in lesbian and bisexual women participating in online mindfulness-based stress reduction by per-protocol analysis.

Within-subject differences	Preprogram, mean (SD)	Postprogram, mean (SD)	12-week follow-up, mean (SD)	Per protocol					
				Within-subject effects, *F* (*df*)	*P* value	Postprogram to preprogram difference, *t* (*df*)	*P* value	Follow-up to preprogram difference, *t* (*df*)	*P* value
Perceived stress^a^	22.73 (4.52)	17.45 (5.80)	18.09 (6.14)	5.29 (2,20)	.01	3.12 (10)	.01	2.49 (10)	.03
Estimated daily heterosexist experiences^b^	1.87 (0.37)	1.65 (0.37)	1.57 (0.33)	4.53 (2,20)	.02	1.90 (10)	.09	4.12 (10)	.002
Discrimination and harassment	1.45 (0.43)	1.23 (0.27)	1.32 (0.52)	2.21 (2,20)	.14	2.30 (10)	.04	1.27 (10)	.23
Family of origin	1.81 (0.90)	1.65 (0.89)	1.58 (1.22)	0.32 (2,20)	.73	0.51 (10)	.62	0.76 (10)	.47
Gender expression	1.50 (0.86)	1.48 (0.73)	1.20 (0.55)	1.69 (2,20)	.21	0.07 (10)	.95	1.89 (10)	.09
HIV/AIDS	1.03 (0.08)	1.02 (0.06)	1.02 (0.06)	1.00 (2,20)	.39	1.00 (10)	.34	1.00 (10)	.34
Isolation	2.32 (0.96)	1.84 (0.88)	1.93 (0.98)	2.67 (2,20)	.09	2.01 (10)	.07	2.23 (10)	.05
Parenting	1.26 (0.44)	1.27 (0.41)	1.20 (0.34)	1.46 (2,20)	.26	−0.29 (10)	.78	1.49 (10)	.17
Victimization	1.00 (0.00)	1.00 (0.00)	1.00 (0.00)	—^c^	—	—	—	—	—
Vigilance	2.24 (1.08)	2.00 (0.86)	1.77 (0.91)	2.57 (1.25)^d^	.13	0.92 (10)	.38	4.20 (10)	.002
Vicarious trauma	4.21 (0.91)	3.36 (1.09)	3.12 (1.29)	4.70 (2,20)	.02	2.69 (10)	.02	3.67 (10)	.004

^a^Total measure scaled 1 to 40, with higher scores equaling higher perceived stress.

^b^Grand and subtotal measures scored 1 to 6, with higher scores equaling greater daily experiences of heterosexism.

^c^Test could not be calculated due to lack of variance in data.

^d^Mauchly’s assumption of sphericity violated; Greenhouse-Geisser correction reported.

**Table 4:** Within-subject differences in perceived and minority stress in lesbian and bisexual women participating in online mindfulness-based stress reduction by intention-to-treat analyses.

Within-subject differences	Preprogram, mean (SD)	Postprogram, mean (SD)	Intention to treat						
			12-week follow-up, mean (SD)	Within-subject effects, *F* (*df*)	*P* value	Postprogram to preprogram difference, *t* (*df*)	*P* value	Follow-up to preprogram difference, *t* (*df*)	*P* value
Perceived stress^a^	24.06 (4.78)	20.44 (6.96)	20.88 (6.96)	4.68 (2,30)	.02	2.77 (15)	.01	2.31 (15)	.04
Estimated daily heterosexist experiences^b^	2.01 (0.40)	1.86 (0.47)	1.80 (0.48)	4.09 (2,30)	.03	1.83 (15)	.09	3.39 (15)	.004
Discrimination and harassment	1.62 (0.81)	1.47 (0.80)	1.53 (0.86)	2.13 (2,30)	.14	2.17 (15)	.047	1.26 (15)	.23
Family of origin	1.90 (1.18)	1.86 (1.19)	1.81 (1.38)	0.32 (2,30)	.72	0.52 (15)	.61	0.76 (15)	.46
Gender expression	1.53 (0.79)	1.52 (0.70)	1.53 (0.86)	1.65 (2,30)	.21	0.07 (15)	.95	1.82 (15)	.09
HIV/AIDS	1.09 (0.21)	1.08 (0.20)	1.08 (0.20)	1.00 (2,30)	.38	1.00 (15)	.33	1.00 (15)	.33
Isolation	2.80 (1.24)	2.47 (1.36)	2.53 (1.38)	2.54 (2,30)	.10	1.93 (15)	.07	2.11 (15)	.05
Parenting	1.27 (0.50)	1.28 (0.48)	1.23 (0.45)	1.44 (2,30)	.25	−0.29 (15)	.77	1.46 (15)	.16
Victimization	1.00 (0.00)	1.00 (0.00)	1.00 (0.00)	—^c^	—	—	—	—	—
Vigilance	2.67 (1.13)	2.50 (1.07)	2.34 (1.18)	2.45 (1.41,21.07)^d^	.12	0.92 (15)	.37	3.42 (15)	.004
Vicarious trauma	4.14 (0.97)	3.56 (1.12)	3.40 (1.29)	4.22 (2,30)	.02	2.47 (15)	.03	3.12 (15)	.007

^a^Total measure scaled 1 to 40, with higher scores equaling higher perceived stress.

^b^Grand and subtotal measures scored 1 to 6, with higher scores equaling greater daily experiences of heterosexism.

^c^Test could not be calculated due to lack of variance in data.

^d^Mauchly’s assumption of sphericity violated; Greenhouse-Geisser correction reported.

**Table 5:** Within-subject differences in perceived and minority stress in gay and bisexual men participating in online mindfulness-based stress reduction, by per-protocol analyses.

Within-subject differences	Per protocol								
	Preprogram, mean (SD)	Postprogram, mean (SD)	12-week follow-up, mean (SD)	Within-subject effects, *F* (*df*)	*P* value	Postprogram to preprogram difference, *t* (*df*)	*P* value	Follow-up to preprogram difference, *t* (*df*)	*P* value
Perceived stress^a^	19.83 (4.53)	12.00 (3.58)	13.33 (4.80)	6.70 (2,10)	.01	3.90 (5)	.01	2.15 (5)	.08
Estimated daily heterosexist experiences^b^	2.31 (0.67)	2.08 (0.38)	1.86 (0.50)	1.33 (2,10)	.31	0.86 (5)	.43	1.22 (5)	.28
Discrimination and harassment	1.89 (0.84)	1.64 (0.61)	2.00 (0.85)	0.64 (2,10)	.55	0.86 (5)	.43	−0.26 (5)	.81
Family of origin	2.72 (0.95)	2.42 (0.48)	2.11 (0.51)	1.90 (2,10)	.20	0.84 (5)	.44	1.87 (5)	.12
Gender expression	1.19 (0.40)	1.17 (0.41)	1.00 (0.00)	0.60 (2,10)	.57	0.12 (5)	.91	1.19 (5)	.29
HIV/AIDS	2.73 (1.40)	2.50 (1.11)	1.90 (1.00)	1.11 (2,10)	.37	0.69 (5)	.52	1.06 (5)	.34
Isolation	3.17 (0.49)	2.75 (1.21)	2.54 (1.30)	0.65 (2,10)	.54	0.63 (5)	.56	0.93 (5)	.39
Parenting	1.06 (0.14)	1.00 (0.00)	1.17 (0.33)	1.00 (2,10)	.40	1.00 (5)	.36	−0.76 (5)	.48
Victimization	1.79 (1.60)	1.70 (1.60)	1.21 (0.33)	0.44 (2,10)	.66	0.42 (5)	.70	0.85 (5)	.44
Vigilance	2.64 (1.28)	2.75 (0.90)	2.08 (0.86)	1.14 (2,10)	.36	−0.44 (5)	.68	0.90 (5)	.41
Vicarious trauma	3.64 (1.56)	3.08 (1.12)	2.78 (0.87)	1.86 (2,10)	.20	1.06 (5)	.34	1.73 (5)	.14

^a^Total measure scaled 1 to 40, with higher scores equaling higher perceived stress.

^b^Grand and subtotal measures scored 1 to 6, with higher scores equaling greater daily experiences of heterosexism.

**Table 6:** Within-subject differences in perceived and minority stress in gay and bisexual men participating in online mindfulness-based stress reduction, by intention-to-treat analyses.

Within-subject differences	Intention to treat								
	Preprogram, mean (SD)	Postprogram, mean (SD)	12-week follow-up, mean (SD)	Within-subject effects, *F* (*df*)	*P* value	Postprogram to preprogram difference, *t* (*df*)	*P* value	Follow-up to preprogram difference, *t* (*df*)	*P* value
Perceived stress^a^	19.88 (3.87)	14.00 (4.81)	15.00 (5.13)	5.27 (2,14)	.02	3.01 (7)	.02	1.99 (7)	.09
Estimated daily heterosexist experiences^b^	2.26 (0.61)	2.09 (0.38)	1.92 (0.48)	1.31 (1.11,2.2)^c^	.29	0.86 (7)	.42	1.20 (7)	.27
Discrimination and harassment	1.81 (0.79)	1.62 (0.60)	1.90 (0.81)	0.65 (2,14)	.54	0.86 (7)	.42	−0.26 (7)	.80
Family of origin	2.85 (1.26)	2.62 (1.17)	2.40 (1.23)	1.82 (2,14)	.20	0.85 (7)	.42	1.77 (7)	.12
Gender expression	1.23 (0.39)	1.21 (0.40)	1.08 (0.24)	0.61 (2,14)	.56	0.12 (7)	.91	1.18 (7)	.28
HIV/AIDS	2.50 (1.26)	2.32 (1.00)	1.88 (0.85)	1.11 (2,14)	.36	0.70 (7)	.50	1.06 (7)	.33
Isolation	3.12 (0.50)	2.81 (1.07)	2.66 (1.15)	0.66 (2,14)	.53	0.64 (7)	.54	0.94 (7)	.38
Parenting	1.10 (0.20)	1.06 (0.18)	1.19 (0.31)	1.00 (2,14)	.39	1.00 (7)	.35	−0.76 (7)	.47
Victimization	1.59 (1.40)	1.34 (0.72)	1.16 (0.30)	0.48 (2,14)	.65	0.42 (7)	.69	0.85 (7)	.42
Vigilance	2.44 (1.16)	2.52 (0.89)	2.02 (0.76)	1.13 (2,14)	.35	−0.45 (7)	.67	0.90 (7)	.40
Vicarious trauma	3.67 (1.44)	3.25 (1.15)	3.02 (1.04)	1.79 (2,14)	.20	1.06 (7)	.32	1.66 (7)	.14

^a^Total measure scaled 1 to 40, with higher scores equaling higher perceived stress.

^b^Grand and subtotal measures scored 1 to 6, with higher scores equaling greater daily experiences of heterosexism.

^c^Mauchly’s assumption of sphericity violated; Greenhouse-Geisser correction reported.

### Gender-Based Comparisons

Per protocol, average perceived stress did not differ by gender at baseline (*t*_15_=−1.26; *P*=.23), postprogram (*t*_15_=−2.08; *P*=.06), or follow-up (*t*_15_*=* −1.64; *P*=.12; Multimedia Appendix 2). ITT analyses indicated that compared with men, women had higher perceived stress at baseline (mean 19.88 vs mean 24.06; *t*_22_=−2.14; *P*=.04), postprogram (mean 14.00 vs mean 20.44; *t*_22_=−2.34; *P*=.03), and follow-up (mean 15.00 vs mean 20.88; *t*_22_=−2.11; *P*=.05).

Per protocol, average daily heterosexist experiences differed by gender; postprogram, women’s average daily heterosexist experiences (mean 1.65, SD 0.37) were lower than men’s (mean 2.08, SD 0.38; *t*_15_=2.27; *P*=.04).

## Discussion

### Principal Findings

OMBSR was feasible and associated with reduced perceived and minority stress among LGB people in Appalachian Tennessee. In terms of feasibility, LGB people logged onto the OMBSR website most days each week to complete readings, videos, and formal and informal mindfulness activities.

Participation in OMBSR was associated with reductions in perceived stress and minority stress for women and in perceived stress among men. In our sample, perceived stress was reduced from baseline by 23% in women and by 40% in men. This is similar to the average clinical and nonclinical presumably heterosexual samples reported by others [14,15]. Participants in Morledge et al’s randomized control study reported a 22% reduction in perceived stress from baseline (mean 22.4) to postintervention (17.2) in a clinical sample. Krusche et al showed a 34% reduction in perceived stress from baseline (mean 23.04) to postintervention (mean 15.05) in a convenience sample. Given the associations between chronic stress and poor health [28-30], the substantial reductions in perceived stress evidenced in our study could have very real and clinically meaningful implications for LGB people residing in Appalachian Tennessee [16].

Women in our study also showed a 12% reduction in minority stress from baseline; however, no changes in minority stress were reported for men. According to a prevailing theory, LGB people experience minority stressors in the form of discrimination and stigma related to their nonheterosexual sexual orientation. These minority stressors are cumulative, exist beyond individual control, and are in addition to daily hassles and stressful life events that are experienced by all people [31,32]. The reductions in minority stress reported for women are important, as minority stress is associated with risky health behaviors and poor physical health outcomes [18,33-38]. In particular, women reported that stress arising from experiences of sexual orientation–related vigilance and vicarious trauma was reduced. Vigilance decreased by approximately 21% from baseline to the 12-week follow-up and vicarious trauma was reduced by 20% between baseline and postprogram assessment and by 26% at the 12-week follow-up.

The changes in vigilance and vicarious trauma may be especially meaningful as they relate to OMBSR. Both concepts, vigilance and vicarious trauma, reflect individuals’ expectations for isolation and negative interactions because of their sexual orientation. Vigilance is the higher arousal and attention regarding the risk for potentially heterosexist and homophobic attitudes and behaviors. Vicarious trauma is the perception of threat for negative interactions and harm because of directly or indirectly witnessing these experiences perpetrated against other LGB women. Both vigilance and vicarious trauma center on a person’s thinking and perceptions about the world around them. OMBSR is designed to change how people think, including thoughts about risk and anticipation of negative interactions. At an individual level, women may not be able to change the real existence of discrimination and harassment arising from heterosexism and homophobia. However, with the help of OMBSR, they may be able to change the way they think about, or anticipate, these negative experiences, thus, reducing stress and the associated deleterious effects of stress on health.

We are not aware of other empirical tests of OMBSR programs on perceived and minority stress among LGB people. However, others have successfully applied mindfulness principles to weight management for lesbian and bisexual women [39]. Our project was among the first to test this question among LGB men and women and to show preliminary evidence of a mainstream behavioral intervention that could reduce the negative consequences of minority stress among LGB women.

Our gender comparison revealed that women and men in our sample were demographically very similar; however, we found evidence of differences in stress. Regarding perceived stress, women reported higher perceived stress at all assessment points and showed a smaller reduction in stress after completing OMBSR, as did men. This may be evidence that lesbian and bisexual women experience confluent and intersectional gender- and sexual orientation–based biases and oppression [17,19,20]. However, compared with men, on average, women reported lower daily heterosexist experiences; they perceived less and were less bothered by daily experiences with heterosexism than men in this study.

Women and men in our sample reported that they felt socially isolated because of their sexual orientation and that a social component overlaid on the existing OMBSR material could enhance their experience. For women, an online social component should allow facilitators and participants to discuss participants’ questions about program activities and increase social connectedness among lesbian and bisexual women. For men, a social component should encourage participants to connect in person to increase accountability to complete daily mindfulness activities and the full 8-week program. This could be facilitated through planned digital or in-person meetings or by encouraging gay and bisexual men to enroll as dyadic pairs, mindfulness teams, with partners or friends. These findings should guide future interventions adaptation and implementation of OMBSR with LGB people in this region.

### Limitations

Our study had limitations. These were pilot studies and lacked control groups; therefore, it is unknown if stress reductions were caused by OMBSR or some unknown external factor. However, we carefully considered this in advance and used a pre-post no comparison design to determine the acceptability and feasibility of OMBSR, which could not have been informed by a controlled condition. We did not set out to test the efficacy of OMBSR in this pilot study, which would require a more rigorous, controlled design. Participants qualitatively reported positive changes in health behaviors (eg, decreased substance use) and outcomes (eg, decreased anxiety); however, these were not measured quantitatively. Future studies should measure self-reported health behaviors and outcomes, as well as anthropomorphic measures of stress (eg, allostatic load) to better understand the health benefits of OMBSR for LGB people.

### Conclusions

OMBSR is a promising stress-reduction intervention that is acceptable to, and feasible for, LGB people in Appalachian Tennessee. LGB participants engaged in OMBSR frequently—completing readings, videos, and informal and formal mindfulness activities most days of the week and with few external prompts. Surprisingly, almost all participants reported that they would not make any changes to the existing OMBSR program content. However, both women and men did suggest modifying the program to reduce social isolation associated with living in rural areas of Appalachian Tennessee.

Owing to their nonheterosexual sexual orientation, LGB people living in rural areas, including Appalachian East Tennessee, experience discrimination and stigma. These minority stressors add to LGB people’s stress in excess of the daily life hassles and stressful events experienced by all people, contributing to poor health. In the absence of comprehensive multilevel interventions that reduce sexual orientation–based discrimination and victimization, individual behavioral–level interventions are necessitated to reduce excess stress among this group. OMBSR is one such solution that is feasible for and acceptable to LGB women and men in Appalachian Tennessee and, as reported qualitatively and via preliminary quantitative data, may improve health in this group.

## Appendix

Multimedia Appendix 1 Demographic characteristics of lesbian, gay, and bisexual women and men participating in an 8-week online mindfulness-based stress reduction program.

Multimedia Appendix 2 Between-subject differences in perceived and minority stress in lesbian, gay, and bisexual women and men participating in online mindfulness-based stress reduction, by per-protocol and intention-to-treat analyses.

## Abbreviations

DEHQ: Daily Experiences with Heterosexism Questionnaire
ITT: intention to treat
LGB: lesbian, gay, and bisexual
MBSR: mindfulness-based stress reduction
OMBSR: online mindfulness-based stress reduction
PSS: perceived stress scale
