# Supplementary material for: A Test of Feasibility and Acceptability of Online Mindfulness-Based Stress Reduction for Lesbian, Gay, and Bisexual Women and Men at Risk for High Stress: Pilot Study
Source: JMIR Ment Health. 2019 Aug 16;6(8):e15048. doi: 10.2196/15048 (PMC6716336; doi:10.2196/15048)
Supplement: Multimedia Appendix 1 [file mental_v6i8e15048_app1.pdf]

Table 2. Demographic characteristics of lesbian, gay, and bisexual women and men participating in an 8-week online mindfulness-based stress reduction program.

	Total participants enrolled in program (intention to treat; n=24)			Participants who completed the program (per protocol; n=17)			Did not complete the program (n=7)		
	Women, n (%)	Men, n (%)	*P*	Women, n (%)	Men, n (%)	*P*	Women, n (%)	Men, n (%)	*P*
Total	16 (67)	8 (33)	—	11 (65)	6 (35)	—	5 (71)	2 (29)	—
Sexual identity	— —		.68			.24			.65
Bisexual	1 (6)	1 (13)		0	1 (17)		1 (20)	0 (0)	
Mostly lesbian/gay/homosexual	3 (19)	1 (13)		2 (18)	0 (0)		1 (20)	1 (50)	
Only lesbian/gay/homosexual	10 (63)	6 (74)		7 (64)	5 (83)		3 (60)	1 (50)	
Other	2 (12)	0 (0)		2 (18)	0 (0)		0 (0)	0 (0)	
Current relationship status			.08			.03			.81
In a committed relationship	11 (69)	2 (25)		9 (82)	1 (17)		2 (40)	1 (50)	
Not in a committed relationship	5 (31)	5 (63)		2 (18)	4 (67)		3 (60)	1 (50)	
Other	0 (0)	1 (12)			1 (17)		0 (0)	0 (0)	

Location of residence			.13			.39			.14
Open country but not on a farm	4 (25)	1 (12)		4 (36)	1 (17)		1 (20)	0 (0)	
On a farm	2 (12)	00 (0)		1 (9)	0 (0)		3 (60)	0 (0)	
Small city or town (under 50,000 residents)	7 (44)	1 (12)		4 (36)	1 (17)		1 (20)	0 (0)	
Medium-sized city (50,000-250,000 residents)	2 (12)	2 (25)		1 (9)	2 (33)		0 (0)	1 (50)	
Suburb near a large city	1 (6)	3 (38)		1 (9)	2 (33)		0 (0)	1 (50)	
Large city (>250,000 residents)	0 (0)	1 (12)		0 (0)	0 (0)		0 (0)	0 (0)	
Type of primary residence			.56			.70			.47
Own house/condominium/town house	6 (43)	4 (50)		4 (40)	2 (33)		2 (50)	2 (100)	
Rent house/condominium/town house	4 (29)	1 (12)		3 (30)	1 (17)		1 (25)	0 (0)	
Rent apartment	1 (7)	2 (25)		1 (10)	2 (33)		0 (0)	0 (0)	
Other	3 (21)	1 (12)		2 (20)	1 (17)		1 (25)	0 (0)	
Educational attainment			.25			.33			.35
High school diploma or General Education Development(GED)	1 (6)	2 (25)		1 (9)	2 (33)		0	0	

Some college, 2-year degree or diploma, technical school	7 (44)	4 (50)		5 (46)	2 (33)		2 (40)	2 (100)	
Bachelor's degree	3 (19)	2 (25)		2 (18)	2 (33)		1 (20)	0 (0)	
Graduate or professional degree	5 (31)	0 (0)		2 (27)	0 (0)		2 (40)	0 (0)	
Employment status			.93			.83			.35
Working full-time for pay	5 (31)	3 (38)		3 (27)	1 (17)		2 (40)	2 (100)	
Working part-time for pay	5 (31)	2 (25)		5 (46)	2 (33)		0 (0)	0 (0)	
Working full-time and part-time for pay	1 (6)	0 (0)		0 (0)	0 (0)		1 (20.0)	0 (0)	
Retired or disabled	4 (25)	2 (25)		2 (18)	2 (33)		2 (40.0)	0 (0)	
Not looking for work, for other reasons	1 (6)	1 (12)		1 (9)	1 (17)		0 (0)	0 (0)	
Hours worked per week for pay			.97			.77			.19
Do not currently work for pay	5 (31)	3 (38)		3 (27)	3 (50)		2 (40)	0 (0)	
1-10 hours/week	1 (6)	0 (0)		1 (9)	0 (0)		0 (0)	0 (0)	
11-20 hours/week	2 (12)	1 (12)		2 (18)	1 (17)		0 (0)	0 (0)	
31-40 hours/week	4 (25)	2 (25)		4 (36)	1 (17)		0 (0)	1 (50)	
41-60 hours/week	4 (25)	2 (25)		1 (9)	1 (17)		3 (60)	1 (50)	
Total household income in US Dollars			.39			.18			.53

Less than US $15,000	2 (12)	1 (12)		0 (0)	1 (17)		0 (0)	0 (0)	
US$15,000-$24,999	4 (25)	0 (0)		4 (36)	0 (0)		2 (40)	0 (0)	
US$25,000-$39,999	2 (12)	3 (38)		1 (9)	2 (33)		1 (20)	1 (50)	
US$40,000-$59,999	1 (6)	0 (0)		1 (9)	0 (0)		0 (0)	0 (0)	
US$60,000-$74,999	4 (25)	1 (12)		4 (36)	1 (17)		0 (0)	0 (0)	
US$75,000 or more	3 (19)	3 (38)		1 (9)	2 (33)		2 (40)	1 (50)	
Amount of total household income provided			.25			.22			.73
None	3 (19)	0 (0)		3 (27)	0 (0)		0 (0)	0 (0)	
Less than half	3 (19)	4 (50)		2 (18)	4 (67)		1 (20)	0 (0)	
About half	2 (12)	2 (25)		1 (9)	1 (17)		1 (20)	1 (50)	
More than half	3 (19)	0 (0)		2 (18)	0 (0)		1 (20)	0 (0)	
All	5 (31)	2 (25)		3 (27)	1 (17)		2 (40)	1 (50)	
Perceived capacity of household income			.69			.40			.79
Not enough to make basic needs	2 (12)	2 (25)		1 (9)	2 (33)		1 (20)	0 (0)	
Enough to meet basic needs	8 (50)	4 (50)		6 (55)	3 (50)		2 (40)	1 (50)	
More than enough to meet basic needs	6 (38)	2 (25)		4 (36)	1 (17)		2 (40)	1 (50)	
