# Supplementary material for: A Test of Feasibility and Acceptability of Online Mindfulness-Based Stress Reduction for Lesbian, Gay, and Bisexual Women and Men at Risk for High Stress: Pilot Study
Source: JMIR Ment Health. 2019 Aug 16;6(8):e15048. doi: 10.2196/15048 (PMC6716336; doi:10.2196/15048)
Supplement: Multimedia Appendix 2 [file mental_v6i8e15048_app2.pdf]

Table 8. Between-subject differences in perceived and minority stress in lesbian, gay, and bisexual women and men participating in online mindfulness-based stress reduction, by per-protocol and intention-to-treat analyses.

	Women, mean (SD)	Men, mean (SD)	*t*	df	*P*	Women, mean (SD)	Men, mean (SD)	*t*	df	*P*
	Per protocol					Intention to treat				
Perceived stress[a]										
Preprogram	22.73 (4.52)	19.83 (4.53)	−1.26	15	.23	24.06 (4.78)	19.88 (3.87)	−2.14	22	.04
Postprogram	17.45 (5.80)	12.00 (3.58)	−2.08	15	.06	20.44 (6.96)	14.00 (4.81)	−2.34	22	.03
Follow-up	18.09 (6.14)	13.33 (4.80)	−1.64	15	.12	20.88 (6.96)	15.00 (5.13)	−2.11	22	.05
Post-Pre Difference	−5.27 (5.61)	−7.83 (4.92)	−0.94	15	.36	−3.62 (5.23)	−5.88 (5.51)	−0.98	22	.34
Follow-up-Pre Difference	−4.64 (6.19)	−6.50 (7.40)	−0.56	15	.59	−3.19 (5.52)	−4.88 (6.94)	−0.65	22	.52
Estimated daily heterosexist experiences[b]										
Preprogram	1.87 (0.37)	2.31 (0.67)	1.79	15	.09	2.01 (0.40)	2.26 (0.61)	1.20	22	.24
Postprogram	1.65 (0.37)	2.08 (0.38)	2.27	15	.04	1.86 (0.47)	2.09 (0.38)	1.18	22	.25
Follow-up	1.57 (0.33)	1.87 (0.50)	1.46	15	.16	1.80 (0.48)	1.92 (0.48)	0.56	22	.58

	Post-Pre Difference	−0.22 (0.38)	−0.23 (0.66)	−0.04	15	.97	−0.15 (0.33)	−0.17 (0.57)	−0.12	22	.91
	Follow-up-Pre Difference	−0.30 (0.24)	−0.45 (0.90)	−0.39	15	.71	−0.21 (0.24)	−0.34 (0.79)	−0.45	22	.66
Discrimination and harassment											
	Preprogram	1.45 (0.43)	1.89 (0.84)	1.18	15	.28	1.63 (0.81)	1.81 (0.79)	0.54	22	.59
	Postprogram	1.23 (0.27)	1.64 (0.61)	1.57	15	.17	1.47 (0.80)	1.63 (0.60)	0.48	22	.63
	Follow-up	1.32 (0.52)	2.00 (0.85)	2.07	15	.06	1.53 (0.86)	1.90 (0.81)	1.00	22	.33
	Post-Pre Difference	−0.23 (0.33)	−0.25 (0.71)	−0.07	15	.94	−0.16 (0.29)	−0.19 (0.61)	−0.17	22	.87
	Follow-up-Pre Difference	−0.14 (0.36)	0.11 (1.07)	0.72	15	.49	−0.09 (0.30)	0.08 (0.90)	0.72	22	.48
Family of origin											
	Preprogram	1.82 (0.90)	2.72 (2.72)	1.93	15	.07	1.98 (1.18)	2.85 (1.26)	1.65	22	.11
	Postprogram	1.65 (0.89)	2.42 (0.48)	1.93	15	.07	1.86 (1.19)	2.63 (1.17)	1.49	22	.15
	Follow-up	1.58 (1.22)	2.11 (0.51)	1.01	15	.33	1.81 (1.38)	2.40 (1.23)	1.01	22	.32
	Post-Pre Difference	−0.17 (1.08)	−0.31 (0.89)	−0.27	15	.79	−0.11 (0.88)	−0.23 (0.77)	−0.31	22	.76

	Follow-up-Pre Difference	−0.24 (1.07)	−0.61 (0.80)	−0.74	15	.47	−0.17 (0.88)	−0.46 (0.73)	−0.86	22	.40
Gender expression											
	Preprogram	1.50 (0.86)	1.19 (0.40)	−0.81	15	.43	1.53 (0.79)	1.23 (0.39)	−1.02	22	.32
	Postprogram	1.48 (0.73)	1.17 (0.41)	−1.15	15	.27	1.52 (0.70)	1.21 (0.40)	−1.40	22	.18
	Follow-up	1.20 (0.55)	1.00 (0.00)	−0.86	15	.40	1.32 (0.86)	1.08 (0.24)	−1.07	22	.30
	Post-Pre Difference	−0.02 (0.74)	−0.03 (0.58)	−0.04	15	.97	−0.01 (0.60)	−0.02 (0.49)	−0.04	22	.96
	Follow-up-Pre Difference	−0.30 (0.53)	−0.19 (0.40)	0.44	15	.67	−0.21 (0.46)	−0.15 (0.35)	0.34	22	.74
HIV/AIDS											
	Preprogram	1.04 (0.08)	2.73 (1.40)	2.96	15	.03	1.09 (0.21)	2.50 (1.26)	3.15	22	.02
	Postprogram	1.02 (0.06)	2.50 (1.11)	3.25	15	.02	1.08 (0.20)	2.33 (1.00)	3.51	22	.01
	Follow-up	1.02 (0.06)	1.90 (1.00)	2.15	15	.08	1.08 (0.20)	1.88 (0.85)	2.63	22	.03
	Post-Pre Difference	−0.02 (0.06)	−0.23 (0.82)	−0.64	15	.55	−0.01 (0.05)	−0.18 (0.70)	−0.65	22	.54
	Follow-up-Pre Difference	−0.02 (0.06)	−0.83 (1.93)	−1.04	15	.35	−0.01 (0.05)	−0.63 (1.67)	−1.03	22	.34

Isolation										
Preprogram	2.32 (0.96)	3.17 (0.49)	2.01	15	.06	2.80 (1.24)	3.13 (0.50)	0.92	22	.37
Postprogram	1.84 (0.88)	2.75 (1.21)	1.79	15	.09	2.47 (1.36)	2.81 (1.07)	0.62	22	.54
Follow-up	1.93 (0.98)	2.54 (1.30)	1.10	15	.29	2.53 (1.38)	2.66 (1.15)	0.22	22	.83
Post-Pre Difference	−0.48 (0.79)	−0.42 (1.63)	0.09	15	.93	−0.33 (0.68)	−0.31 (1.39)	0.03	22	.98
Follow-up-Pre Difference	−0.39 (0.57)	−0.63 (1.64)	−0.34	15	.74	−0.27 (0.50)	−0.47 (1.42)	−0.39	22	.70
Parenting										
Preprogram	1.26 (0.44)	1.06 (0.14)	−1.40	15	.19	1.27 (0.50)	1.10 (0.20)	−1.16	22	.26
Postprogram	1.27 (0.41)	1.00 (0.00)	−2.21	15	.05	1.28 (0.48)	1.06 (0.18)	−1.61	22	.12
Follow-up	1.20 (0.34)	1.17 (0.33)	−0.18	15	.86	1.23 (0.45)	1.19 (0.31)	−0.24	22	.82
Post-Pre Difference	0.02 (0.17)	−0.06 (0.14)	−0.86	15	.40	0.01 (0.14)	−0.04 (0.12)	−0.89	22	.38
Follow-up-Pre Difference	−0.06 (0.13)	0.11 (0.36)	1.44	15	.17	−0.04 (0.11)	0.08 (0.31)	1.46	22	.16
Victimization										

	Preprogram	1.00 (0.00)	1.79 (1.60)	1.21	15	.28	1.00 (0.00)	1.59 (1.40)	1.20	22	.27
	Postprogram	1.00 (0.00)	1.46 (1.60)	1.38	15	.23	1.00 (0.00)	1.34 (0.72)	1.35	22	.22
	Follow-up	1.00 (0.00)	1.21 (0.33)	1.54	15	.19	1.00 (0.00)	1.16 (0.30)	1.49	22	.18
	Post-Pre Difference	0.00 (0.00)	−0.33 (1.97)	−0.42	15	.70	0.00 (0.00)	−0.25 (1.67)	−0.42	22	.68
	Follow-up-Pre Difference	0.00 (0.00)	−0.58 (1.69)	−0.85	15	.44	0.00 (0.00)	−0.44 (1.45)	−0.85	22	.42
Vigilance											
	Preprogram	2.24 (1.08)	2.64 (1.28)	0.68	15	.51	2.67 (1.13)	2.44 (1.16)	−0.46	22	.65
	Postprogram	2.00(0.86)	2.75 (0.90)	1.69	15	.11	2.50 (1.07)	2.52 (0.89)	0.05	22	.96
	Follow-up	1.77 (0.91)	2.08 (0.86)	0.68	15	.50	2.34 (1.18)	2.02 (0.76)	−0.70	22	.49
	Post-Pre Difference	−0.24 (0.88)	0.11 (0.62)	0.87	15	.40	−0.17 (0.73)	0.08 (0.53)	0.86	22	.40
	Follow-up-Pre Difference	−0.47 (0.37)	−0.56 (1.51)	−0.18	15	.86	−0.32 (0.38)	−0.42 (1.30)	−0.20	22	.85
Vicarious trauma											
	Preprogram	4.21 (0.91)	3.64 (1.56)	−0.97	15	.35	4.15 (0.97)	3.67 (1.44)	−0.97	22	.34

	Postprogram	3.36 (1.09)	3.08 (1.12)	−0.50	15	.62	3.56 (1.12)	3.25 (1.15)	−0.64	22	.53
	Follow-up	3.12 (1.29)	2.78 (0.87)	−0.58	15	.57	3.40 (1.29)	3.02 (1.04)	−0.71	22	.48
	Post-Pre Difference	−0.85 (1.04)	−0.56 (1.28)	0.51	15	.62	−0.58 (0.94)	−0.42 (1.11)	0.38	22	.70
	Follow-up-Pre Difference	−1.09 (0.99)	−0.86 (1.22)	0.42	15	.68	−0.75 (0.96)	−0.65 (1.10)	0.24	22	.81

[a]Total measure scaled 1 to 40, with higher scores equaling higher perceived stress.

[b]Grand and subtotal measures scored 1 to 6, with higher scores equaling greater daily experiences of heterosexism.
